# Blockade of co-inhibitory receptor immune checkpoint protein TIM3/CD366 augments the anti-cancer activity of CAR-T therapy in solid tumors: An ovarian cancer example

**DOI:** 10.1016/j.gendis.2025.101978

**Published:** 2025-12-11

**Authors:** Binglan Zhang, Fuping Zhu, Song Wang, Yong Huang, Shishi Yu, Shaorong Tian, Minmin Li, Pan Li, Qian Xue, Bingqiang Zhang

**Affiliations:** a Department of Gastroenterology, The First Affiliated Hospital of Chongqing Medical University, Chongqing 400016, China; b Department of Gastroenterology, University-Town Hospital of Chongqing Medical University, Chongqing 401331, China; c Department of Hepatobiliary Surgery, The Ninth People's Hospital of Chongqing, Chongqing 400700, China; d Department of Biotherapy, State Key Laboratory of Biotherapy and Cancer Center, West China Hospital, Sichuan University, Chengdu, Sichuan 610041, China; e Department of Gastroenterology, Chongqing Key Laboratory of Translational Research for Cancer Metastasis and Individualized Treatment, Chongqing University Cancer Hospital, Chongqing 400030, China; f Department of Oncology, The First Affiliated Hospital of Chongqing Medical University, Chongqing 400016, China

**Keywords:** Cell therapy, Chimeric antigen receptor, Immunotherapy, Inhibitory checkpoint, TIM-3

## Abstract

Strategies that enhance the function of chimeric antigen receptor-modified T (CAR-T) cells for solid tumors are critical. Inhibitory immuno-checkpoints blockade could potentially enhance CAR-T cell function. TIM-3 is an important negative regulator of T cell activity, but whether TIM-3 blockade could affect CAR-T cell function remains unclear. In our study, we successfully constructed TIM-3-silenced CAR-T cells by dual-promoter lentivirus vectors that simultaneously express the TIM-3 targeting short hairpin RNA (shRNA) and a third-generation CAR recognizing HER2. We demonstrated that down-regulation of TIM-3 did not affect the phenotype of CAR-T cells. CAR-T cells with TIM-3 blockade exhibited higher lytic cytotoxicity to target cells *in vitro*. Additionally, TIM-3-silenced CAR-T cells displayed robust anti-tumor activity in a murine xenograft model, which is comparable to standard CAR-T cells. Our study demonstrates the effect of down-regulation of immune checkpoint TIM-3 on the anti-tumor function of CAR T cells, providing new ideas for improving the potency of CAR-T cell therapies in solid tumors.

## Introduction

Chimeric antigen receptor-modified T (CAR-T) cell therapy has exhibited potent anti-tumor activity against hematological malignancies in recent years.^1^, ^2^, ^3^ However, the efficacy for solid tumors is still not ideal. CAR-T cell therapies face several challenges, including a lack of ideal targets, inefficient trafficking infiltration, immunosuppressive tumor microenvironment, and associated toxicity.^4^ Among these, a critical barrier against the use of CAR-T cells for treating solid tumors is the immunosuppressive tumor microenvironment, especially inhibitory immuno-checkpoints.^5^, ^6^, ^7^

T cell immunoglobulin-3 (TIM-3), also called CD366 or HAVCR2, is a co-inhibitory receptor that expresses on interferon-gamma (IFN-γ)-producing T cells, CD4^+^FoxP3^+^ Treg cells, and other innate immune cells, including macrophages and dendritic cells.^8^ It has been reported that TIM-3 can negatively regulate T cell responses through promoting the development of CD8^+^ T cell exhaustion and inducing expansion of myeloid-derived suppressor cells.^9^^,^^10^ TIM-3 is not just restricted to immune cells, which is also expressed on various cancer cells, such as melanoma, hepatocellular carcinoma, clear cell renal cell carcinoma, and malignant pleural mesothelioma.^11^, ^12^, ^13^, ^14^ Recent studies have reported that the expression of TIM-3 on cervical cancer cells can promote tumor invasion and metastasis through different mechanisms, including facilitating tumor cell migration and invasion.^15^ Therefore, the overexpression of TIM-3 in the tumor microenvironment is considered to be one important factor leading to tumor immune escape.

It has been fully reported that the combination of CAR-T cell therapy with antibody targeting inhibitory checkpoint programmed death-1 (PD-1), or the expression knockdown or other intrinsic blockades of PD-1 on the CAR-T cells, has exerted an enhanced anti-tumor activity.^16^, ^17^, ^18^, ^19^ However, Koyama et al have reported that anti-PD-1 treatment can lead to up-regulation of TIM-3 at treatment failure, and TIM-3 antibody addition could overcome the resistance to PD-1 blockade.^20^ Simultaneously, multinomial studies have shown that a combination of monoclonal antibodies against TIM-3 and PD-1 was found to be more effective in controlling tumor growth.^21^^,^^22^ In addition, many clinical trials are underway to explore the use of anti-TIM-3 antibodies either alone or in combination with anti-PD-1 in both solid and hematologic cancers (ClinicalTrials.gov). Thus, TIM-3 blockade may be a new strategy to restore immune function and a promising method to improve CAR-T cell function.

In this study, rather than combining CAR-T cells with inhibitory checkpoint TIM-3 antibody treatment, we constructed dual-promoter lentivirus vectors that simultaneously express the TIM-3 targeting short hairpin RNA (shRNA) and a third-generation CAR recognizing HER2. Then, we evaluated whether down-regulation of TIM-3 could enhance the anti-tumor potential of CAR-T cells with specificity to HER2 (HER2 CAR-T) *in vitro* and *in vivo*. Finally, we demonstrated that HER2 CAR-T cells that secrete a TIM-3-shRNA scFv had a greater lytic cytotoxicity to target cells, and the adoptive transfer of TIM-3 silencing CAR-T cells significantly delayed tumor growth in the early days *in vivo*. However, the anti-tumor effect was reduced at the later stage of animal experiments.

## Materials and methods

### Cell lines

Human cervical cancer cell line HeLa, lentivirus packaging cell line HEK 293TD, and human ovarian cancer cell line SKOV3 were purchased from American Type Culture Collection (Manassas, Virginia, USA) and cultured in Dulbecco's modified Eagle's medium (Invitrogen, Grand Island, New York) supplemented with 10% heat-inactivated fetal bovine serum.

### CAR construction and lentivirus production

Five different TIM-3 targeting shRNA sequences were purchased from Sigma, USA. HER-2-specific CAR was provided by the State Key Laboratory of Biotherapy, Sichuan University (China). The most effective TIM-3 targeting shRNA sequence was first screened out, and then was inserted into the downstream of the U6 promoter. Scramble shRNA sequence was constructed in the same manner as the control. HER-2-specific CAR sequence was inserted into the downstream of the EF1a promoter. Finally, the HER-2-specific CAR and TIM-3 targeting shRNA sequence were inserted into a PCLK lentiviral vector (Addgene, Cambridge, Massachusetts). The whole sequence was confirmed by direct sequencing. Lentiviruses were produced in HEK-293 TD cells using the calcium phosphate method. The detailed procedures for the packaging of lentivirus are the same as in our previous article.^23^ The concentrated lentivirus titers were determined by quantitative real-time PCR.

### T cell culture

All the experiments were approved by the Ethics Committee of the First Affiliated Hospital of Chongqing Medical University (China). Human peripheral blood mononuclear cells were isolated from healthy voluntary donors. After centrifugation by Ficoll-Hypaque density gradients (Sigma–Aldrich), these cells were cultured in a X–VIVO 15 medium (Lonza) containing 2.5% human AB serum (Sigma–Aldrich) and 100 U/mL cytokines rhIL-2 (Novoprotein). To improve the culture effect, human peripheral blood mononuclear cells were often stimulated with anti-CD3 and anti-CD28 magnetic beads immediately after centrifugation (Gibco, Thermo Fisher, Waltham, Massachusetts, USA).

### Lentivirus transduction

The isolated human peripheral blood mononuclear cells described above were activated for 24 h to selectively stimulate T cells. Then, 1 mL RetroNectin (50 μg/mL) was added to the 6-well cell culture plates for overnight incubation at 4 °C. The following day, RetroNectin solution was aspirated, and phosphate-buffered saline solution plus 2% bovine serum albumin was added to the wells for 30-min incubation at room temperature. The wells were then washed twice with phosphate-buffered saline solution. The concentrated PCLK-CAR-TIM-3 targeting shRNA-encoding lentivirus or PCLK-CAR-encoding lentivirus was diluted and added to each RetroNectin-coated well. The 6-well plates were centrifuged at 1000 *g* at 32 °C for 2 h. After centrifugation, the activated T cells were added to each well for further incubation. 48 h later, T cells were replaced with new fresh medium and expanded for the next experiment.

### Flow cytometry

The CAR expression on T cells and the phenotype of T cells were examined using flow cytometry. All operations were performed in accordance with the manufacturer's recommended protocols. To detect CAR scFv, the above modified T cells were stained with PE-conjugated anti-F(ab)2 (Jackson ImmunoResearch, USA). The phenotype of CAR-T cells was evaluated by staining with anti-CD3, anti-CD4, anti-CD8, anti-CD62L, and anti-CD45RO antibodies (Biolegend, USA). Flow cytometry data were analyzed by Flow Jo software 7.6.

### Cytotoxicity and coculture assays

Firstly, we co-cultured CAR-T cells or untreated T cells with Galectin-9^+^ or Galectin-9^–^ SKOV3 tumor cells at a different effector cell: target cell ratio (E:T ratio = 5/10) in 96-well plates. The supernatants were then collected at 24 h for further analysis. Enzyme-linked immunosorbent assay (ELISA) was used to detect IFN-γ and tumor necrosis factor alpha (TNF-α) secretion (eBioscience) in accordance with the manufacturer's instructions.

### Mouse xenograft model

All animal studies were performed in accordance with the animal health-care regulations of Chongqing Medical University. Female NOD/SCID BALB/c mice (all 5–6 weeks of age) were obtained from the Beijing HFK Bioscience Co. Ltd. (HFK). To establish the tumor model, mice were first injected with 2 × 10^6^ SKOV3-LUC tumor cells (SKOV3 tumor cell line stably expressing luciferase) intraperitoneally. Seven days after inoculation, tumor-bearing mice were randomized into three groups receiving untreated T cells, HER-2-specific CAR-T cells, or HER-2-specific TIM-3 silencing CAR-T (CAR-T kdTim-3) cells. CAR-T cells were administered at a dose of 2 × 10^6^ cells/mouse via intraperitoneal injection. Bioluminescent imaging for tumors was performed on scheduled days using an *in vivo* imaging system.

### RNA sequencing

On day 60, the purified RNA from tumor-infiltrating T cells in CAR-T kdTim-3 and HER-2-specific CAR-T group was shipped on dry ice to the Beijing Genomics Institute (China) for analysis. Quantile normalization and subsequent data processing were performed using the Dr. Tom multi-omics data Mining System. Differentially expressed genes were identified through fold-change filtering. The transcriptional profiles of differentially expressed genes were used for visualization by heatmap through z-score normalization. Deep-sequencing analyses, including Gene Ontology (GO) enrichment analysis, Kyoto Encyclopedia of Genes and Genomes (KEGG) pathway enrichment analysis, and gene set variation analysis (GSVA), were performed based on the differentially expressed genes.

### Statistical analysis

Statistical analysis was performed using GraphPad Prism 5.0 (GraphPad Software, Inc., San Diego, California, USA). All the data were expressed as mean ± standard deviation. One-way ANOVA was used to compare three groups. Statistical analysis for IFN-γ and TNF-α secretion was performed using a paired Student's *t*-test. Statistical significance was defined as *P*-values <0.05.

## Results

### Construction of TIM-3 silencing CAR

**Figure 1:**
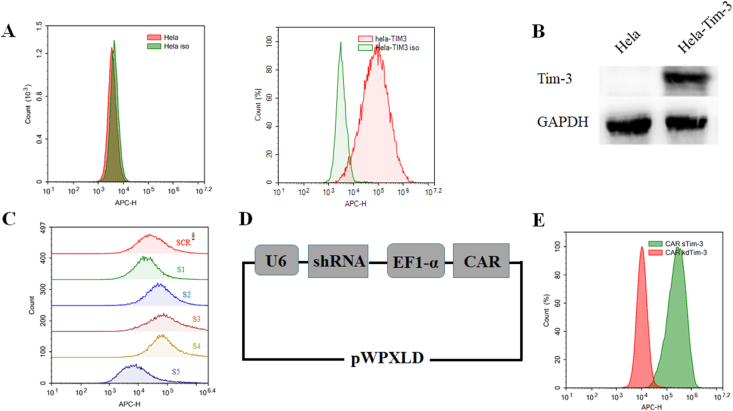
Construction of TIM-3-silenced chimeric antigen receptor (CAR). **(A)** HeLa cells were first transduced with lentivirus encoding TIM-3. Results of the flow cytometry assay to detect the expression of TIM-3 in the infected HeLa cells. **(B)** Results of Western blotting assay to detect the protein expression of TIM-3 in the infected HeLa cells. **(C)** HeLa-Tim-3 cells were transduced with lentiviruses encoding different TIM-3 shRNA sequences, and the expression of TIM-3 in HeLa-Tim-3 cells was tested. **(D)** Schematic diagram of TIM-3 silencing CAR, which consists of the signal peptide of U6 promoter, TIM-3 shRNA, EF1-α promoter, HER2-targeting CAR, and pWPXLD lentiviral vector. **(E)** The expression of TIM-3 in HeLa-Tim-3 cells was tested. TIM-3 silencing CAR was called CAR kdTim-3. CAR sTim-3 was used as a control.

Since TIM-3 is rarely expressed in tumor cells, HeLa cells were transduced with lentivirus encoding TIM-3 first to achieve the stable expression for the next experiment. Two days later, purinomycin was added to the cells for 7 days. After screening, the transduction efficiency was determined by flow cytometry and Western blotting. As shown in Figure 1A, TIM-3 was expressed in more than 90% of HeLa cells by flow cytometry. The result of Western blotting further showed that TIM-3 was successfully stably expressed in HeLa cells (HeLa-Tim-3) (Fig. 1B).

Five different shRNA sequences targeting TIM-3 were synthesized to screen for valid ones, and a scramble sequence (SCR) was used as a control. Hela-Tim-3 cells were transduced with lentiviruses encoding different TIM-3 shRNA sequences. The TIM-3 silencing efficiency was analyzed by flow cytometry to exclude invalid shRNA sequences. Finally, we screened out one efficient TIM-3 shRNA sequence, shRNA-5 (Fig. 1C).

We firstly constructed a third-generation CAR targeting HER2, composed of the signal peptide of human IL-2 (Sp of hIL-2), anti-HER2 scFv, CD8α-hinge, CD8 transmembrane domain (CD8α-TM), and intracellular signaling motifs of CD28, 4-1BB, and CD3ζ domain. Then, a dual-promoter vector was constructed, which could express TIM-3 targeting shRNA and CAR simultaneously. U6 promoter was used to initiate the expression of shRNA, and the expression of HER2-targeting CAR (CAR HER2) was driven by elongation factor 1-alpha (EF1-α) promoter. Finally, the shRNA-5 and CAR HER2 were inserted into a pWPXLD lentiviral vector, called CAR kdTim-3 (Fig. 1D). Simultaneously, SCR and CAR HER2 were also constructed as above, called CAR sTim-3. To verify the silencing effect of the new vector CAR kdTim-3, HeLa-Tim-3 cells were transduced with lentiviruses encoding CAR kdTim-3 or CAR sTim-3. As shown in Figure 1E, the silencing effect of CAR kdTim-3 was further confirmed.

### Generation of TIM-3 silencing CAR-T cells

**Figure 2:**
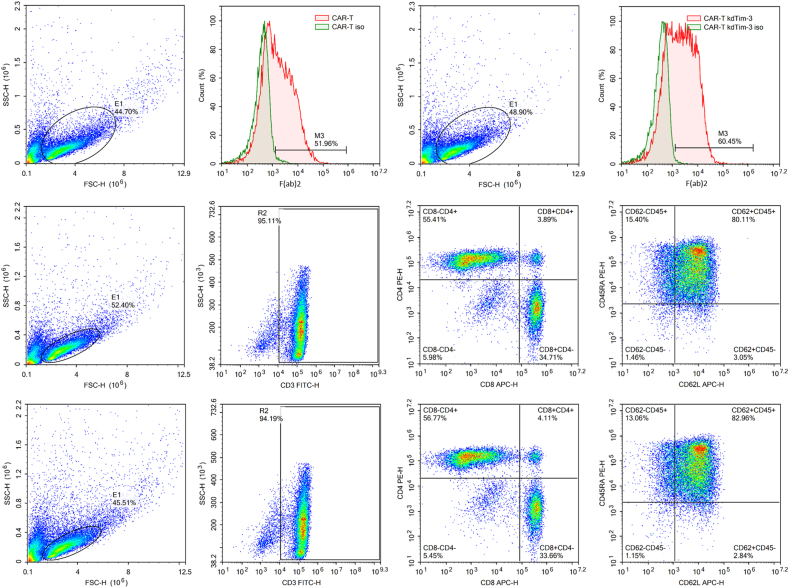
Expression of chimeric antigen receptor (CAR) on T lymphocytes and the phenotypes of CAR-T cells. F(ab)2 subset is the proportion of T cells expressing CAR. **(A, B)** Flow cytometry analysis of the CAR expression on the modified HER2-specific CAR-T cells (A) and TIM-3-silenced HER2-specific CAR-T cells (B). **(C, D)** Representative expression of CD3, CD4, CD8, CD45RO, and CD62L markers on HER2-specific CAR-T cells (C) and TIM-3-silenced HER2-specific CAR-T cells (D) by flow cytometry. Numbers represent percentages of cells per quadrant.

Human peripheral blood mononuclear cells were isolated from the peripheral blood of healthy donors, stimulated with anti-CD3/CD28 immune magnetic beads for 1 day, and then transduced with lentiviruses encoding TIM-3 targeting shRNA and HER2-specific CAR (CAR kdTim-3) or HER2-specific CAR alone. The transduction efficiency was determined by staining with anti-F(ab)2 in flow cytometry after a week. Lentiviral transduction of T cells with CAR kdTim-3 vector displayed efficient induction of CAR expression, approximately 50%–60%, which was equivalent to conventional HER2-specific CAR (Fig. 2A and B).

On day 10 post-transduction, the phenotype of CAR-T cells was analyzed. As depicted in Figure 2C, CD3 was expressed by more than 95% of HER2-specific CAR-T cells, while CD8-positive and CD4-positive cells accounted for 55.41% and 34.71%, respectively. The phenotype of CAR-T kdTim-3 cells was similar to that of HER2-specific CAR-T cells. Of these, the CD3-positive subset accounted for 94.19% of the total number of CAR-T kdTim-3 cells, while CD8-positive and CD4-positive cells were 56.77% and 33.66%, respectively (Fig. 2D). In addition, we further identified the type of memory T cells. The major part of the CAR-T kdTim-3 cells were central memory T cells, which was also similar to that of HER2-specific CAR-T cells (Fig. 2C and D). In conclusion, the above result revealed that TIM-3 silencing did not affect the phenotype of CAR T cells.

### Down-regulation of TIM-3 enhances the specific cytotoxicity of CAR-T cells

**Figure S1:**
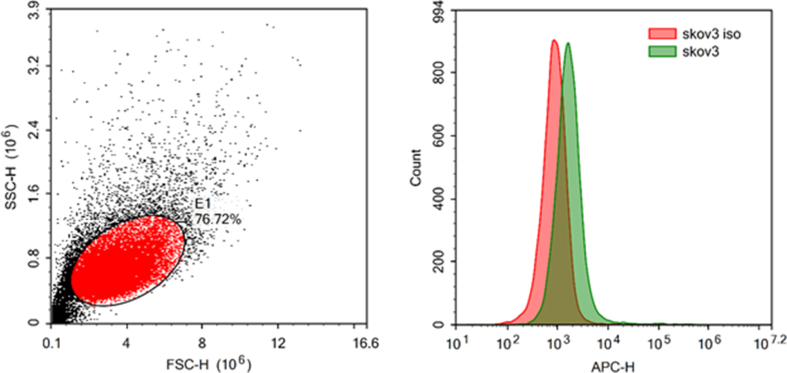
Expression of the Galectin-9 molecule in SKOV3 tumor cells. SKOV3 cells were first transduced with lentivirus encoding Galectin-9. Results of flow cytometry assay to detect the expression of Galectin-9 in the infected SKOV3 cells.

Galectin-9 is a specific ligand of TIM-3, and the combination of the two can mediate T cell immune tolerance. However, Galectin-9 is not expressed in tumor cells. To better verify the killing efficiency of CAR-T kdTim-3 cells *in vitro*, Galectin-9 was first overexpressed on the cell membrane surface of SKOV3 tumor cells by lentivirus encoding Galectin-9 (Fig. S1).

**Figure 3:**
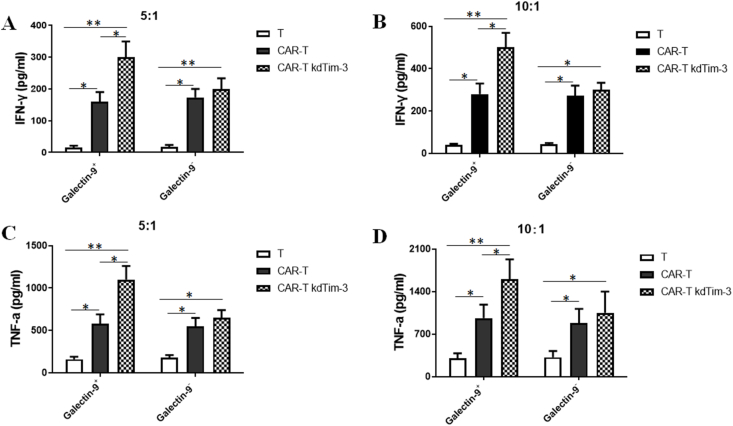
Specific IFN-γ and TNF-α release of T lymphocytes transduced with TIM-3-silenced HER2-specific chimeric antigen receptor (CAR) or HER2-specific CAR. **(A, B)** TIM-3-silenced CAR-T cells and control T cells were co-incubated with Galectin-9^+^ or Galectin-9^–^ SKOV3 tumor cells (E:T ratio 5:1 or 10:1). At 20 h after coculture, a specific enzyme-linked immunosorbent assay was used to analyze the supernatant for IFN-γ cytokine-release. Results were presented as mean ± standard deviation. **(C, D)** The detection of TNF-α in the same culture supernatant. Results were presented as mean ± standard deviation. ∗*P* < 0.05 and ∗∗*P* < 0.01.

To investigate whether TIM-3 silencing is capable of specifically promoting the HER2-specific CAR-T cells recognizing tumor cells, we co-incubated HER2-specific CAR-T kdTim-3 cells with Galectin-9^+^ or Galectin-9^–^ SKOV3 tumor cells (E:T ratio = 5/10). Untreated T cells and HER2-specific CAR-T cells were used as controls. 20 h later, the amount of secreted effector cytokines, including IFN-γ and TNF-α, was determined by ELISA assay. The results showed that HER2-specific CAR-T kdTim-3 cells could specifically recognize the Galectin-9^+^ SKOV3 tumor cell and secrete high-dose IFN-γ (Fig. 3A and B) and TNF-α (Fig. 3C and D) when compared with that of HER2-specific CAR-T cells. For Galectin-9-negative SKOV3 tumor cells, there was no significant difference in IFN-γ and TNF-α levels between the CAR-T kdTim-3 cells and CAR-T cells (Fig. 3). In addition, for Galectin-9^+^ or Galectin-9^–^ SKOV3 tumor cells, we observed higher IFN-γ and TNF-α induction in the CAR-T groups compared with the untreated T cells group.

### The effect of TIM-3 silencing on the anti-tumor function of CAR-T cells *in vivo*

**Figure 4:**
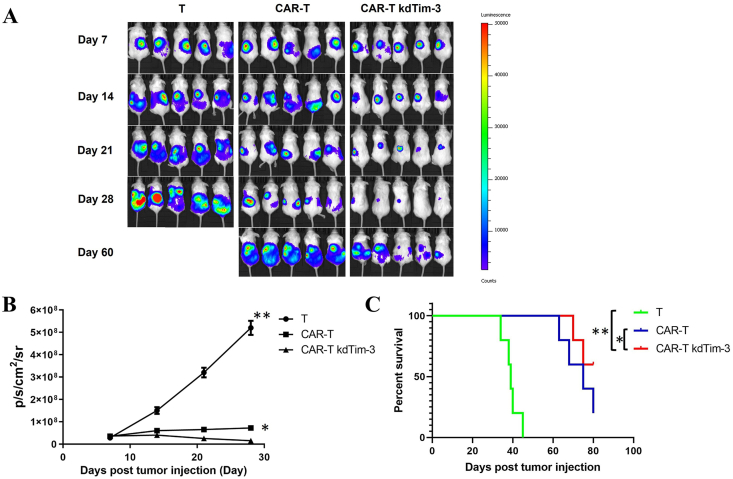
TIM-3 silencing augmented the anti-tumor activity of chimeric antigen receptor-T (CAR-T) cells *in vivo*. 2 × 10^6^ SKOV3 tumor cells expressing luciferase were intraperitoneally inoculated in a xenograft mouse model, and 7 days after inoculation, the 2 × 10^6^ HER2-specific CAR-T kdTim-3 cells or CAR-T cells, or untreated T cells were intraperitoneally administered. **(A, B)** Tumor growth was monitored using an *in vivo* imaging system. **(C)** Survival curve of 80-day post-treatment. ∗*P* < 0.05 and ∗∗*P* < 0.01.

To further evaluate the anti-tumor function of the TIM-3 silencing CAR-T cells, we employed SKOV3 as a xenograft tumor model. In this xenograft model, 2 × 10^6^ SKOV3-luc cells were inoculated intraperitoneally 1 week before CAR-T infusion. Tumor growth was monitored using an *in vivo* spectrum imaging system. For CAR-T kdTim-3 treatment, 2 × 10^6^ sorted TIM-3-silenced HER2-specific CAR-T cells cultured for 10 days were administered by intraperitoneal injection, and HER2-specific CAR-T cells and non-infected T cells were used as controls. In the early days (within a month), treatment with CAR-T kdTim-3 cells significantly inhibited tumor growth compared with control groups. Tumor size was substantially smaller in all mice treated with CAR-T kdTim-3 cells, whereas progressive tumor growth was observed in the control group of mice treated with CAR-T cells or non-infected T cells. However, the anti-tumor effect was significantly reduced at the later stage. On day 60, the tumor burden of mice treated with standard HER2-specific CAR-T and CAR-T kdTim-3 cells was similar (Fig. 4A and B). The survival of mice that received CAR-T kdTim-3 cells or control T cells was shown in Figure 4C.

**Figure 5:**
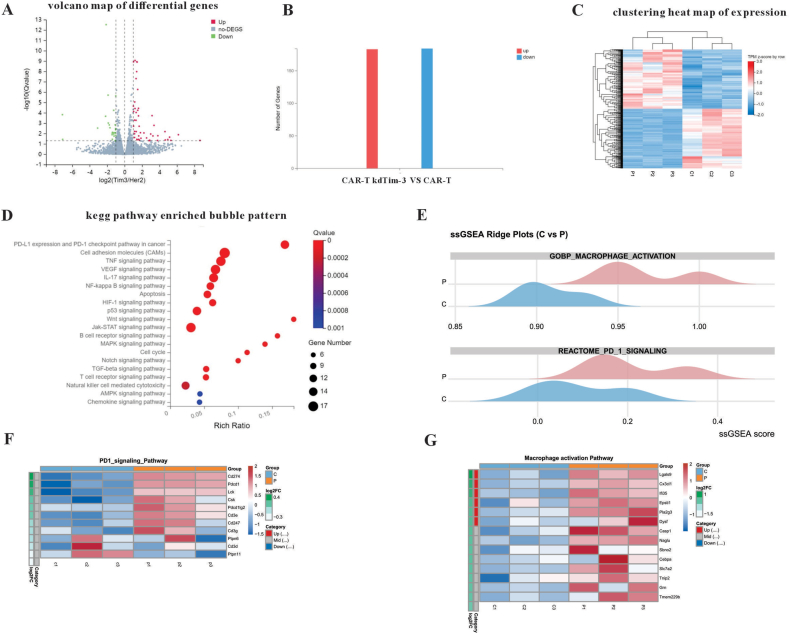
TIM-3 silencing induced unique gene expression in human HER2-specific chimeric antigen receptor-T (CAR-T) cells. **(A)** Volcano plots of HER2-specific CAR-T kdTim-3 cells versus CAR-T cells. Each dot represents a differentially expressed gene. The red dots indicate up-regulated differentially expressed genes (DEGs) that passed the screening threshold, green dots indicate down-regulated DEGs, and black dots indicate nonsignificant DEGs. **(B)** Statistically differentially expressed genes. **(C)** Heat map of DEGs. **(D)** Gene Ontology (GO) enrichment analysis of DEGs. **(E)** Gene set variation analysis (GSVA) of the genes related to TIM3 functions, including the PD-1 signaling pathway and macrophage activation pathway. Genes with significant changes in the PD1 signaling pathway and macrophage activation pathway were shown in **(F)** and **(G)**. C, control group (CAR-T cells); P, experimental group (CAR-T kdTim-3 cells).

To understand the mechanisms of the influence of TIM-3 silencing on the HER2 CAR-T cells, we compared the gene expression profiles between the CAR-T kdTim-3 cells and HER2-specific CAR-T cells isolated from tumor tissues, respectively. It was found that TIM-3 silencing profoundly altered the transcriptional profile of the CAR-T cells, with 183 genes up-regulated and 184 genes down-regulated (Fig. 5A and B). Heat map and GO enrichment analysis of differentially expressed genes were shown in Figure 5C and D. We performed GSVA of the genes related to TIM3 functions, including PD-1 signaling pathway, macrophage activation pathway, and Th1 differentiation. Among these, the significant changes are shown in the PD1 and macrophage signaling pathways. Relevant results were shown in Figure 5E–G.

## Discussion

Previous studies have revealed that CAR-T cell therapy has remarkable successes in patients with hematological malignancies; however, its efficacy in the treatment of solid tumors was compromised. Recently, it has been demonstrated that CAR-T cell therapy and PD-1 checkpoint blockade are a rational combination in solid tumor models.^19^^,^^24^, ^25^, ^26^, ^27^, ^28^ Building on this success, efforts are ongoing to extend it toward the immune checkpoints in the immunosuppressive tumor microenvironment. TIM-3 emerges as an attractive target for immune modulation in cancer immunotherapy.^29^ Numerous studies have shown that antibodies against TIM-3 are very effective for treating various cancers.^21^^,^^30^, ^31^, ^32^, ^33^ Based on results from previous findings, we hypothesized that the combination of TIM-3 checkpoint blockade with CAR-T cell therapy could improve the therapeutic efficiency for solid tumors.

In this study, we engineered HER2-specific TIM-3-silenced CAR-T cells to treat ovarian cancer and compared the anti-tumor activity of CAR-T cells to the TIM-3-silenced CAR-T cells. It has been reported that the blockade efficacy of the antibody is short-lived and relies upon repeated administration.^19^^,^^34^ Previously, several studies have practiced the strategy of intrinsic blockade of PD-1 with shRNA or CRISPR/Cas9 technologies.^19^^,^^26^^,^^27^^,^^35^^,^^36^ To directly counteract TIM-3-mediated inhibition, we used lentiviral vectors to combine CAR with a TIM-3 shRNA and achieved efficient transfection. Systemic administration of checkpoint blockade can result in immune-related adverse events.^37^, ^38^, ^39^ Immune-related adverse events are one of the major problems that limit checkpoint blockade-based immunotherapy. In our study, delivery of the TIM-3-blocking HER2-scFv is primarily localized to the site of the tumor. Therefore, we hypothesize that this strategy may decrease the immune-related adverse events associated with systemic checkpoint blockade.

Adoptive cell therapy, the infusion of T cells to patients, represents a promising approach for the treatment of advanced metastatic cancers. The phenotype of T cells is closely related to the anti-tumor ability. Central memory T cells (T_CM_) are the most important type of tumor-specific effector cells, playing the main role in anti-tumor activity, and may be the most appropriate type for adoptive cell therapy.^40^, ^41^, ^42^ It has been reported that loss of PD-1 can alter memory T cell content and generation.^43^ However, CRISPR-Cas9 disruption of PD-1 led to a selective enrichment of T_CM_ for CAR-T cells.^27^^,^^28^ Our data also indicated that TIM-3 silencing did not affect the phenotype of CAR T cells, and central memory T cells were still the major part of the TIM-3-silenced CAR-T cells (Fig. 2).

Previous studies have demonstrated that the blockade of TIM-3 by monoclonal antibody has achieved excellent anti-tumor effect.^30^, ^31^, ^32^ Recently, Lee et al have reported that anti-TIM-3 CAR-T cell therapy could improve the clinical outcomes of patients with acute myeloid leukemia.^44^ There are only a few reports that have analyzed the effects of Tim3 knockdown on anti-tumor CAR-T cell functions.^45^^,^^46^ Our current study further demonstrated that down-regulation of TIM-3 could augment the anti-tumor activity of CAR-T cells via enhancing cytotoxicity of CAR-T cells to targeted cells (Fig. 3). Galectin-9 is a ligand of TIM-3, and the combination of the two can mediate T cell immune tolerance. The expression level of Galectin-9 in tumor cells is extremely low. It is mainly expressed in immune cells, such as T cells, macrophages, dendritic cells, and non-immune cells (*e.g.*, endothelial cells and epithelial cells). In *in vitro* assays, only tumor cells and CAR-T cells were present. To better verify the killing efficiency of CAR-T kdTim-3 cells, Galectin-9 was first overexpressed on the cell membrane surface of SKOV3 tumor cells by lentivirus encoding Galectin-9. To evaluate TIM-3-silenced HER-2-specific CAR-T cell potential *in vivo*, the SKOV3 model with high expression levels of HER-2 was adopted. When conducting *in vivo* animal experiments, it is the real tumor environment with various immune cells involved, and tumor cells do not need specially to express galectin-9 separately. Our study showed that TIM-3-silenced HER-2 CAR-T cells significantly delayed tumor growth initially, but the anti-tumor effect was significantly reduced at the later stage (Fig. 4). One possible reason may be the limited persistence of CAR-T cells in solid tumors. The results of preclinical and clinical studies have indicated that the transport efficiency of CAR-T cells to solid tumors is very low after intravenous injection, and most of this transport may occur shortly after injection. The limited data obtained from human studies indicate that the few CAR-T cells entering solid tumors have limited persistence and do not proliferate widely.^47^^,^^48^ In addition, the result of GSVA showed that multiple genes within the CAR-T cells undergo expression changes in the later stage of Tim-3 silencing, and the most significant is the PDL1/PD-1 signaling pathway. Therefore, CAR-T cells with dual silencing of immune checkpoints PD-1 and Tim-3 may be a new strategy for improving the therapeutic efficacy of CAR-T cell therapy for solid tumors in the future. Further verification will be conducted in our subsequent research.

In conclusion, our study found that TIM-3 checkpoint blockade could only temporarily enhance the anti-tumor activity of CAR-T cells. The potential advantages of TIM-3-silenced CAR-T cells require further study, and our study provides a new insight for improving the efficacy of CAR-T cells in treating solid tumors.

## CRediT authorship contribution statement

**Binglan Zhang:** Writing – original draft, Visualization, Validation, Supervision, Software, Resources, Project administration, Methodology, Investigation, Funding acquisition, Formal analysis, Data curation, Conceptualization. **Fuping Zhu:** Software, Resources, Methodology, Investigation, Formal analysis, Data curation. **Song Wang:** Software, Resources, Methodology, Investigation, Formal analysis, Data curation. **Yong Huang:** Software, Resources, Methodology, Investigation, Formal analysis, Data curation. **Shishi Yu:** Resources, Project administration, Methodology, Investigation, Formal analysis. **Shaorong Tian:** Resources, Methodology, Formal analysis. **Minmin Li:** Software, Resources, Methodology. **Pan Li:** Software, Resources, Methodology. **Qian Xue:** Software, Resources, Methodology. **Bingqiang Zhang:** Writing – review & editing, Visualization, Validation, Supervision, Project administration, Methodology, Investigation, Formal analysis, Data curation, Conceptualization.

## Ethics declaration

This study was approved by the Ethics Committee of the First Affiliated Hospital of Chongqing Medical University (No. 2024-209-01). We declare written informed consents were obtained from all participants in the manuscript. All animal experiments were approved by the Institutional Animal Care and Use Committee of Chongqing Medical University. All contributing authors agree to the publication of this article.

## Funding

This work was supported by the 10.13039/501100001809National Natural Science Foundation of China (No. 81703057/H1611) and Chongqing Natural Science Foundation (China) (No. CSTB2022NSCQ-MSX0934).

## Conflict of interests

The authors declared no conflict of interests.
